# Complete mitochondrial genome of *Saccharina* cultivar ‘Hainong No.1’ (*Saccharina japonica* × *latissima*)

**DOI:** 10.1080/23802359.2017.1383197

**Published:** 2017-09-25

**Authors:** Na Liu, Jing Zhang, Cui Liu, Yue Li, Tao Liu, Shan Chi

**Affiliations:** a College of Marine Life Sciences, Ocean University of China, Qingdao, China;; b College of Biology Engineering, Qilu University of Technology, Jinan, China;; c Qingdao Haida BlueTek Biotechnology Co., LTD, Qingdao, China

**Keywords:** *Saccharina cultivar*, Hainong No.1, mitogenome, phylogenetic analysis, parental identification

## Abstract

Here, we sequenced the complete mitogenome of ‘Hainong No.1’, a hybrid *Saccharina* cultivar produced by crossing the cultivars ‘Zaohoucheng’ (♂) and ‘Pingbancai’ (♀). Circular mapping revealed that the mitogenome was 37,657 bp in length and had an overall AT content of 64.66%, including 35 protein-encoding genes, three ribosomal RNA genes (rRNA), 25 transfer RNA genes (tRNA) and three open reading frames (ORF). A phylogenetic tree constructed from the amino acids separated the six cultivars into two groups; ‘Hainong No.1’ had a closer evolutionary relationship with the cultivars ‘Zaohoucheng’, ‘Pingbancai’ and ‘Ailunwan’, whereas ‘Rongfu’ and ‘Fujian’ formed a distinct cluster. Further comparison between the ‘Hainong No.1’ and the parental mitogenomes displayed that ‘Pingbancai’ and ‘Zaohoucheng’ were the female and male parents, respectively.

*Saccharina* (Phaeophyceae, Laminariales) is an economically important macroalgal in China that is highly valued for use in foodstuffs and industrial raw materials of extractions of iodine, algin, mannitol among others (Jensen 1993; Kawashima 2004). Hybridization is now one of the most effective ways to breed the new *Saccharina* cultivars of high quality, high yield, and higher stress tolerance.

The surface of ‘Hainong No.1’, a *Saccharina* cultivar produced by a ‘Zaohoucheng’ × ‘Pingbancai’ cross, features brown granular spots and prominently exhibits characteristics of early maturity. The mitogenomes of 22 *Saccharina* species are available in the NCBI database, including the three cultivars ‘Rongfu’ (JF937591) (Zhang, Li, et al. 2011), ‘Ailunwan’ (KU556731) (Zhang Liu, et al. 2016), and ‘Pingbancai’ (KX073817) (Zhang, Gao, et al. 2016). Here, we presented the complete mitogenome of *Saccharina* cultivar ‘Hainong No.1’(specimen number: 2013071810, collected from Rongcheng, Shandong Province, China (37°15’39”N, 122°33’56”E), and stored at −80 °C in the Culture Collection of Seaweed at the Ocean University of China) via the homologous PCR amplification method described by (Zhang, Liu, et al. 2011). The protein-coding and ribosomal RNA genes were annotated based on full mitogenomes of *Saccharina japonica* (NC_013476) and ‘Pingbancai’, and the transfer RNA genes were identified using tRNAscan-SE v.1.21 software (Lowe and Eddy 1997). The phylogenetic analysis was performed with MrBayes v.3.0 software.

The complete mitogenome of ‘Hainong No.1’, when mapped, composed a circular molecule of 37,657 bp (GenBank accession number MF622087), with a nucleotide composition of 28.48% A (10,841), 14.73%C (5,605), 20.61% G (7,843) and 36.18% T(13,767), and had an overall AT content of 64.71%. The mitogenome encoded 35 protein-coding genes, three rRNA genes (23S, 16S and 5S), three ORFs (orf41, orf130 and orf377), and 25 tRNA genes that were scattered throughout the entire genome. The coding region consisted of 28,969 bp, accounting for 76.9% of total genome length. All protein-coding genes started with an ATG codon, and terminated with TAA, TAG and TGA codons. In addition, all the tRNA genes were encoded on an H-strand and most likely possessed clover-leaf-shaped secondary structures. Gene types and numbers showed a high level of conservation when compared to previously published mitogenomes of other *Saccharina* cultivars.

Six closely related breeding varieties for which complete mitogenome sequences were available (‘Hainong No.1’, ‘Zaohoucheng’, ‘Pingbancai’, ‘Ailunwan’, ‘Rongfu’ and ‘Fujian’), as well as *S. japonica* and *Saccharina longissima*, were selected to construct a Bayesian phylogenetic tree. Phylogenetic analysis was based on the polymorphisms of the amino acid sequences of 16 shared protein-encoding genes (including *rpl6*, *rps2*, *rps4*, *nad1*, *tatC*, *rpl16*, *atp6*, *nad2*, *cox1*, *nad9*, *cob*, *cox2*, *nad4*, *nad5*, *nad6*, *rps10*), with *Laminaria digitata* used as the outgroup. The results indicated that six cultivars were divided into two branches (Figure 1), and that ‘Hainong No.1’ had a closer evolutionary relationship with the cultivars ‘Zaohoucheng’, ‘Pingbancai’ and ‘Ailunwan’, all of which belonged to the *S. japonica* lineage; ‘Rongfu’ and ‘Fujian’ formed a separate cluster due to the presence of unique variant sites absent in *S. japonica*. Our phylogenetic tree also provided further support for the current interpretation of the genetic relationships among *Saccharina* cultivars.

**Figure 1.**
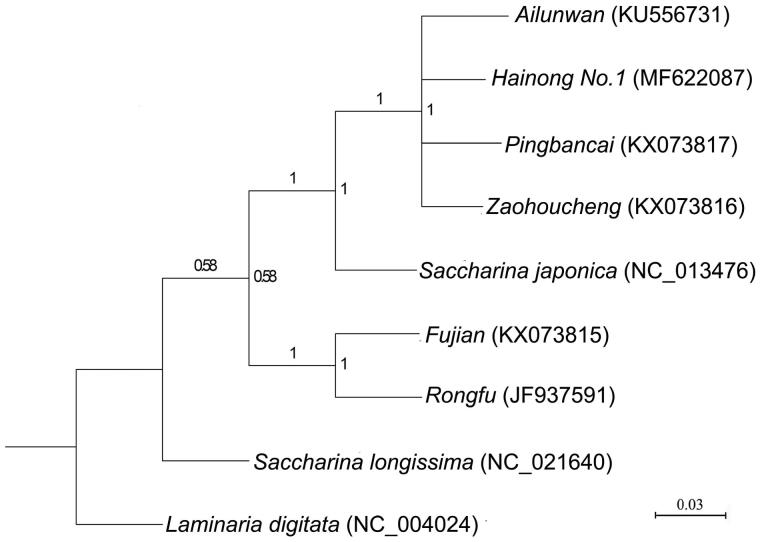
Phylogenetic tree derived from the Bayesian analysis and constructed based on polymorphisms of the amino acid sequences of 16 mtDNA protein-encoding genes.

Mitogenome analysis is a maternally inherited model that aids in identifying parental sources. A comparison of the complete ‘Hainong No.1’ mitogenome with those of its parents revealed a high degree of similarity among the three, except that the 25th and 81st nucleotide sites (C, A) of the *nad6* gene in ‘Hainong No.1’ were the same (C, A) as those of ‘Pingbancai’ as opposed to those of ‘Zaohoucheng’ (T, C). From these results, we concluded that ‘Pingbancai’ is the female parent of ‘Hainong No.1’ and ‘Zaohoucheng’ is the male parent, and confirmed the previous speculation regarding parental identification.
